# Randomized control trial of high fidelity vs low fidelity simulation for training undergraduate students in neonatal resuscitation

**DOI:** 10.1186/s13104-015-1623-9

**Published:** 2015-11-03

**Authors:** Archana Nimbalkar, Dipen Patel, Amit Kungwani, Ajay Phatak, Rohitkumar Vasa, Somashekhar Nimbalkar

**Affiliations:** Department of Physiology, Pramukhswami Medical College, Karamsad, Anand, Gujarat 388325 India; Department of Pediatrics, Pramukhswami Medical College, Karamsad, Anand, Gujarat; Central Research Services, Charutar Arogya Mandal, Karamsad, Anand, Gujarat 388325 India; Division of Neonatology, Department of Pediatrics, University of Chicago Medical Center and Mercy Hospital and Medical Center, Chicago, USA

**Keywords:** Simulation, Resuscitation, Neonate, Students

## Abstract

**Background:**

Knowledge acquisition and skill maintenance are important in learning neonatal resuscitation. Traditionally this is taught by using low fidelity mannequins. Technological advancement enabled a move towards high fidelity mannequins. In a low resources setting, it is incumbent to ensure reasonable cost benefit ratio before investing in technology.

**Methods:**

A randomized control trial was conducted in 101 undergraduate students who were assigned to conventional Resusci^®^ Baby Basic or SimNewB group over a period of 3 days. The lectures were the same for both groups but the hands on training was on different mannequins. There were five experienced and accredited teachers who were standardized for training the students. Both the groups received a written test and a Megacode before and after the training, and 3 months later a post-test.

**Results:**

The baseline written exam score (p = 0.07), Megacode assessment score (p = 0.19) and sex distribution (p = 0.17) were similar in both groups. Both groups showed significant improvement in the written exam score as well as in the Megacode assessment score at post-test and 3 months (retention) period. However there was no significant difference in the “improvement” between both the groups with respect to written exam (p = 0.38) or Megacode assessment (p = 0.92). Further the post-test and 3 month scores were comparable for the skills as well as content components suggesting that the skills were retained in 3 months with an opportunity of self learning them.

**Conclusions:**

Due diligence is a caveat before contemplating the acquisition of high fidelity mannequins by educational centers for neonatal resuscitation.

## Background

Neonatal resuscitation requires the acquisition of cognitive, technical, and behavioral skills. The traditional Neonatal Resusitation Program (NRP), until 2010, had been predominantly based on didactic sessions with additional skills station training. Since 2010, emphasis has been placed on behavioral skills, and simulation methodology fulfills this void. “Simulation is a technique, not a technology, to replace or amplify real experiences with guided experiences, often immersive in nature, that evoke or replicate substantial aspects of the real world in a fully interactive fashion” [1]. The Apollo 13 rescue mission and Skylab 2 repairs used simulation to improve efficacy [2]. Simulation has a large role to play in the education of professionals in industries where there is an inherent risk of significant error and where real-life training is costly and/or dangerous [3]. Medical simulators not only provide life-like models of actual patients; they also involve the most advanced forms of information technology, which allows for repetitive standardized training in various invasive procedures, decision making processes, and human interactions [4].

SimNewB was introduced in 2009, which is an interactive high fidelity simulator for neonatal skills training designed by Laerdal, in conjunction with the American Academy of Pediatrics (AAP), to meet the training requirements of the NRP. SimNewB accurately represents a full-term, 50th percentile newborn female, and measures approximately 50 cm in length and weighs about 3.5 kgs. The fidelity required for a particular application depends on the specific goal. Complex training aids are not required where learners are learning the basic skills involved in a task [5]. Simulation based learning strategies have a favorable impact on self efficacy and motivation for learning that affects acquisition of clinical skills as well as knowledge. These can be integrated into teaching methodologies to promote active learning [6].

While meta-analysis of studies done in simulation for resuscitation show that it is highly effective, they still call for more research in use of simulation as an educational tool [7, 8]. High fidelity simulation has several benefits. It provides video recording of trainees’ performance, thus making debriefing and learning easier. The simulation also allows for repetitive practice, utilizing different scenarios with various levels of difficulty. High fidelity simulators are expensive and setting up expensive simulation labs in a resource-challenged country such as India can be quite challenging. There is an increasing focus on simulation in the recently revised newborn resuscitation guidelines, making it a central part of the training. A recent systematic review on high fidelity mannequins for advanced life support training showed moderate benefits for improving skill performance. It also showed no significant benefit beyond course completion [9]. None of the studies included have been done in India and hence with a large population of medical students and new-borns, it is needed that high fidelity simulation be explored in this setting, especially considering the fact that high fidelity mannequins may not be accesible to the students that may require them.

A simulation course built into the curriculum of 3rd year medical students demonstrated a significant favorable impact on clinical management skills and leadership skills. It is expected that such a curriculum will enhance the ability to manage acute clinical problems which can increase with increased exposure to simulation [10].

The aim of our study was to compare the acquisition and retention of neonatal resuscitation skills, particularly cognitive and technical skills, acquired through use of high fidelity vs low fidelity simulation training.

## Methods

The study was approved by the institutional Human Research Ethics Committee.

Instrument: Written test of 40 questions and Megacode of AAP. The written test was derived from the questions placed in the NRP textbook of AAP. The Megacode is a validated instrument developed by the AAP which tests the psychomotor and cognitive skills of resuscitating a newborn [11]. The Megacode has a 20 item checklist with a 3 (0, 1 and 2) point scale and includes 5 critical skills, none of which should be missed.

Trainers: Four of the five trainers were trained on the SimNewB simulator in the preceding year and had used SimNewB in the intervening duration. The resource people were certified trainers in neonatal resuscitation based on the NRP guidelines by the AAP. Each had a minimum of 2 years of experience as a NRP trainer.

Sample size: Neonatal resuscitation is a mandatory part of curriculum for final year medical students. All 103 students were included as the study subjects. Formal power analysis for calculation of sample size was not done prior to the study. However a sample of size 44 per group was sufficient to detect a moderate effect size of 0.60 between the groups at 5 % alpha error and 80 % power.

Informed consent was obtained from all participants.

Conduct of training: The study was conducted over a period of 3 days using the SimNewB (High Fidelity Simulator) of Laerdal Inc. and the low fidelity Resusci^®^ Baby Basic of Laerdal Inc. Students in groups of 40 individuals on day 1, 40 on day 2, and then 23 on day 3 were randomized to the high fidelity simulator or the low fidelity Resusci^®^ Baby Basic on each day. Randomization was done by WinPepi software by a statistician not involved in the study into high fidelity (HiFi) and low fidelity (LoFi) groups. The first 40 numbers were administered the intervention on day 1 as per their allocated intervention. NRP books of the AAP were provided to all of the MBBS students on the day of the course. At the start of each day, a pre-test of 40 questions and an advanced Megacode to the students was administered. This was followed by a 3 h didactic session of nine lectures based on the NRP textbook of the AAP. Following that, on each day, students were split into two groups. Both groups received training on preparation for resuscitation, initial steps, bag and mask ventilation, and endotracheal intubation at skill stations for a period of about 3 h. Students had little theory and practical exposure in neonatal resuscitation until they entered in the study, which eliminated possible confounding factors. Both the groups had an equal opportunity to finish the scenarios. The students in LoFi group were able to finish scenarios about 30 min earlier. Videotaping and debriefing was not done for either of the groups due to organizational limitations. Case based scenarios were used for both groups.

The HiFi group had the advantage of using several high fidelity features such as cry of mannequin, breathing effort, breath sounds, grunts, cyanosis, movements of limbs, saturations and heart rate displays, gastric tube insertion, cutting umbilical cord, etc.

Following this, a written post-test and Megacode assessment was done for the HiFi group on the simulator and for the LoFi group on the conventional Laerdal mannequin. A student scoring more than or equal to 32 of 38 marks and correctly demonstrating the 5 critical skills was considered ‘pass’. Analysis was done to look for differences between the groups for skills and knowledge acquisition. After 3 months, we conducted a repeat written test and Megacode evaluation to check for retention of skills and knowledge. At the end of 3 months following the course, the trainees completed a questionnaire regarding their experience with didactic portion of the course, simulation methodology, conduct of the course, instructor performance, etc.

### Statistical analysis

Descriptive statistics [mean (SD), frequency (%)] was used to depict the characteristics of the study population. Paired *t* test was applied to test inter-group differences. Independent sample t-test on difference score/Chi-square test was applied to test for difference in skills and knowledge acquisition between the groups depending on the type of variable. Though the study participants were randomized, the teachers who assessed them were not blinded. The statistician was provided the data as group 1 and group 2 rather than high fidelity and low fidelity. The group identity was decoded after the analysis and interpretation was complete. Statistical significance was considered if p-value was less than 0.05.

## Results

All the 103 student trainees in their final year of medical school were randomized using a balanced randomization technique in order to ensure an equal number of participants in both groups (52 in low fidelity group and 51 in high fidelity group). One student from each group did not show up for the training. At the 3 month follow up (retention) evaluation, 46 from low fidelity group and 48 from high fidelity group were present.

A total of 73 (72.3 %) males and 28 (27.7 %) females participated in the study. The gender distribution (Male) [40 (78.4 %) vs 33 (66.0 %)], the mean (SD) pre-test written score [18.87 (6.06) vs 21.04 (5.80)] and the mean (SD) Megacode score [9.75 (6.16) vs 11.38 (6.27)] were similar between the groups (Table 1). Not a single participant from either group passed the Megacode test before the training. One participant from low fidelity group and five participants from high fidelity group performed the five main steps of Megacode correctly before the training.

**Table 1:** Baseline characteristics and within and between group comparisons of low fidelity vs high fidelity training

	Low fidelity (N = 51)	High fidelity (N = 50)	p value for between group comparisons using difference scores
Gender, frequency (%)			
Male	40 (78.4)	33 (66.0)	
Written, mean (SD)			
Pre-test	18.87 (6.06)	21.04 (5.80)	0.39
Post-test	33.90 (3.28)	35.16 (2.67)	
p-value for within group comparison (pre vs post)*	*<0.001*	*<0.001*	
Post 3 months (retention) test	32.43 (3.33)	32.91 (3.57)	
Megacode (score)			
Pre-test	9.75 (6.16)	11.38 (6.27)	0.92
Post-test	29.67 (5.57)	31.46 (6.14)	
p-value for within group comparison (pre vs post)*	*<0.001*	*<0.001*	
Post 3 months (retention) test	30.15 (5.56)	29.25 (6.17)	
Megacode results (post training), frequency (%)			p-value for independence of attributes**
Pass	35 (68.6)	38 (76.0)	0.41
Re-evaluate	16 (31.4)	12 (24.0)	

Italic values indicate statistically significant differences

*p-values were generated using paired t-test

**p-value was generated using chi-square test

**Fig. 1:**
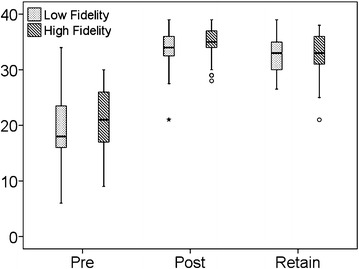
*Box Plot* depicting comparison of written test scores

**Fig. 2:**
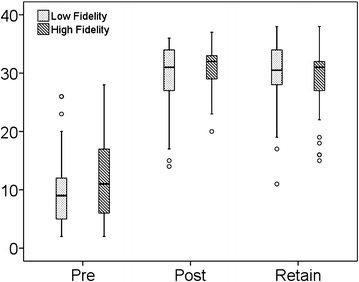
*Box Plot* depicting comparison of Megacode scores

A statistically significant improvement in the post-test score was noted in both the written and Megacode tests for each of the groups but this improvement was similar across groups. The post-test Megacode result was similar in both groups (p = 0.41) (Table 1). The 3 months test scores revealed that the students in both the groups retained the knowledge without any significant difference between the groups. In general, the training worked and both training methodologies had a similar impact (Figs. 1, 2).

Feedback was obtained from the students at the time of retesting for retention of technical and cognitive skills at 3 months following the post-test.

While most of the students found the lectures helpful, 12/46 (26.09 %) students from low fidelity group and 9/47 (19.15 %) students from high fidelity group reported the lectures to be not helpful.

Bag and mask ventilation, routine care and initial steps were perceived as the most important topics, as well as the most helpful lectures. About 60 % of the students from both groups felt that they would remember more than 60 % of the content of these lectures in the future (Table 2).

**Table 2:** Feedback of students regarding contents of lectures

		Low fidelity (N = 46), frequency (%)	High fidelity (N = 47), frequency (%)
Were the lectures helpful?	Yes	34 (73.91)	38 (80.85)
	No	12 (26.09)	9 (19.15)
Name the Most helpful lectures?	Bag and mask ventilation	14 (30.43)	16 (30.04)
	Initial steps	10 (21.74)	13 (27.66)
	Routine care	10 (21.74)	9 (19.15)
	Endotracheal intubation	5 (10.87)	3 (6.38)
	Administration of drugs	2 (4.35)	2 (4.25)
	Chest compression	1 (2.17)	3 (6.38)
	Nothing	4 (8.70)	1 (2.13)
What part of the course do you think is the most important?	Bag and mask resuscitation	14 (30.43)	13 (27.66)
	Routine care	12 (26.09)	16 (34.04)
	Initial steps	9 (19.57)	14 (29.79)
	Endotracheal intubation	8 (17.39)	2 (4.26)
	Chest compressions	3 (6.52)	2 (4.26)
How much of the course do you think you will remember in terms of (%)	<20 %	0 (0)	2 (4.26)
	>80 %	10 (21.74)	8 (17.02)
	20–40 %	1 (2.17)	2 (4.26)
	40–60 %	18 (39.13)	1 (29.79)
	60–80 %	17 (36.96)	2 (44.68)

The number of students who preferred to be in the other group was significantly higher in the LoFi [22 (47.83 %)] group as compared to HiFi [12 (25.53 %)] group (p = 0.04). More students from HiFi [9 (19.15 %)] group wanted additional practice as compared to LoFi [2 (4.35 %)] group but the difference was not statistically significant (p = 0.06). This may be because of the mannequin which was utilized. However, in terms of the skills acquired and the confidence to assist or handle newborn emergencies in resuscitation, both groups fared similarly. Twenty (21.5 %) students were not confident in handling newborn emergencies in resuscitation independently but most [88 (94.6 %)] were confident to assist a senior person in such a situation. Forty (43.0 %) students perceived neonatal resuscitation to be very important in their medical career (in terms of handling and caring for ill/asphyxiated neonates) (Table 3).

**Table 3:** Feedback regarding perception of the course (conduct of the course, instructor etc.)

		Low fidelity (N = 46), frequency (%)	High fidelity (N = 47), frequency (%)
Did you get enough time to practice?	No	2 (4.35)	9 (19.15)
	Yes	44 (95.65)	38 (80.85)
Would you have preferred to be in another group?	No	24 (52.17)	35 (74.47)
	Yes	*22 (47.83)*	*12 (25.53)*
Were the instructors helpful?	No	2 (4.35)	1 (2.13)
	Yes	44 (95.65)	46 (97.87)
Do you think that you will be able to independently take care of a newborn with difficulty of breathing at birth?	May be	0 (0)	1 (2.13)
	No	11 (23.91)	9 (19.15)
	Yes	35 (76.09)	37 (78.72)
Do you think that this course improves your ability to help a more skilled person during the resuscitation of a newborn with breathing difficulty?	No	2 (4.35)	3 (6.38)
	Yes	44 (95.65)	44 (93.62)
Would you be interested in doing the course again?	No	18 (39.13)	22 (46.81)
	Yes	28 (60.87)	25 (53.19)
Would you recommend this course to your juniors?	No	2 (4.35)	2 (4.26)
	Yes	44 (95.65)	45 (95.74)
In terms of learning neonatal resuscitation how useful would it be to you in your medical career (in terms of helping babies)?	<5 %	0 (0)	1 (2.13)
	*>85* *%*	*14 (30.43)*	*8 (17.02)*
	05–20 %	2 (4.35)	2 (4.26)
	20–50 %	12 (26.09)	17 (36.17)
	50–70 %	11 (23.91)	8 (17.02)
	*70–85* *%*	*7 (15.22)*	*11 (23.40)*

Italic values indicate statistically significant associations

## Discussion

We present our experience with two simulation methodologies, low and high fidelity. Many similar studies recruited between 15 and 53 participants [12–16]. Our study represents one of the largest numbers of participants, i.e. 101 undergraduate medical students. To our knowledge, this is the first report of use of a high fidelity neonatal simulator and the first study comparing the efficacy of high fidelity simulation with low fidelity simulation for neonatal resuscitation training for undergraduate medical students in India.

Our study results indicate that magnitude of improvement in skills acquisition is not significantly different with high fidelity simulation vs low fidelity simulation and that the level of short term retention of these skills is also not different between the two groups. In a study on 39 undergraduate students, it was found that all students of 4 groups (only lecture, lectures + videos, lectures + low fidelity and lecture + high fidelity) performed equally well in the written test for knowledge of resuscitation. However in the skills testing, only lecture group performed badly, while the other three groups did not differ from each other. It was thus concluded that high fidelity simulation is of no great benefit to the newer students [17]. The no significant differences between the groups might be due to the fact that newer students had training related to common scenarios and hence the immersion required to experience simulation was similar.

The SimNewB is a relatively newer form of technology, and the training was carried out by instructors, freshly trained. Hence it is possible that all of the features of the SimNewB were not fully utilized. Additionally, we could not achieve full environmental simulation which might under deliver the goods.

We found no decline in skills at 3 months after training which is unusual. If an integrated learning course is taught over a period of 2–3 years there is more likelihood of retention until the completion of internship and beyond [18]. The absence of decline in skills at 3 months in the current study can be ascribed to the fact that these students were close to their final graduation examination and the 3 months assessment was done less than 2 months before the graduation assessment. The graduation examination is of great importance and includes a station on neonatal resuscitation for undergraduates. It is quite possible that the students would have studied and discussed the allotted study material as well as practiced skills for the graduation examination. It is well known that discussions around case scenarios are a powerful tool to retain knowledge as well as psychomotor skills [19]. Hence their results were quite similar after 3 months.

Campbell et al. [12] randomized 15 residents to either a high fidelity (SimBaby) or a traditional plastic mannequin (ALS Baby). Similar to our students, the SimBaby residents rated the experience on high fidelity simulator better than the traditional plastic mannequin, and required less redirection from instructors compared to the residents using plastic mannequin during the Megacode. Campbell et al. demonstrated no difference in the written evaluation scores or the performance task times between the two groups. Based on their study and assuming a type I error of 0.05 and a power of 80 %, about 100 residents would have been required [12]. Our study included 101 undergraduate students. Similar results were also demonstrated in a study conducted by King et al. [13], who randomized 49 nursing students to either static or high-fidelity simulation. There were no significant differences observed between the groups on written examination; however the high-fidelity group outperformed the static simulation group on Megacode performance. Hoadley et al. [14] randomized 53 health care providers to low vs high technical fidelity Advanced Cardiac Life Support training. No significant effect on resuscitation knowledge or skills was observed; however the participants subjectively rated the high fidelity experience favorably.

In a similar study by Cavaleiro et al. [20], 45 final year undergraduate students were randomized to self-study and high fidelity simulator groups. The objective was to compare 30 min supervised self-study vs 30 min neonatal resuscitation session using high fidelity Gaumard simulator; however there were no differences between the pre-test and post-test study scores or between groups. The self-study group had no simulator sessions and was exposed to only the theoretical aspects of resuscitation. Also the pre-test and the post-test did not evaluate any kind of psychomotor skills, which are required in resuscitation.

In yet another study with 1454 students, comparing simulation-based training versus video-based training of anesthesia scenarios, there was no difference in the post-test scores between the two groups. They also noticed that the participants enjoyed/appreciated the simulation session much more compared to the alternative method. Retention of skills was not evaluated [21].

While there is considerable literature on use of simulation in resuscitation, there are few randomized controlled trials that have evaluated neonatal resuscitation. Two systematic reviews that have evaluated such studies have not found a significant benefit of using simulation training for neonatal resuscitation. However the reviews have been hampered by the differing methodologies of studies included and low sample size of most studies [22, 23]. Even more crucial is whether clinical outcomes are influenced by simulation training. Innovative methodologies are needed to study improvement in clinical outcomes.

Considering very high neonatal mortality and morbidity in India, ‘Essential Newborn Care’ (resuscitation at birth, thermoregulation, prevention of infection, breastfeeding and feeding of low birth weight neonates) is taught in the final year of graduation. The findings of this study may be contextual to developing economies with similar training pattern. The high confidence perceived by the students may not necessarily imply clinical competency. Further the feedback was taken after 3 months which may have introduced a recall bias, albeit we expect a mature feedback (without ‘heat of moment’) by prolonging the time point of the same.

We recommend that if the goals of training are to emphasize technical and cognitive aspects of neonatal resuscitation skills, then high fidelity simulators do not offer any added advantage for new students. Considerations should also be given to videotaping, debriefing, and observing teamwork/communication/leadership skills of the trainees; as these are powerful tools and may have added benefits.

## Conclusions

High fidelity simulation is an useful tool for teaching neonatal resuscitation to undergraduate students. Students are comfortable with such technology and have improved self-confidence after the training. However it does not demonstrate any added advantage over low fidelity simulation for neonatal resuscitation even after 3 months.

## Abbreviations

NRP: Neonatal Resuscitation Program
AAP: American Academy of Pediatrics
HiFi: high fidelity
LoFi: low fidelity
